# Laser Deposition of Metal Halide Perovskites

**DOI:** 10.1021/acsenergylett.4c01466

**Published:** 2024-08-01

**Authors:** Tatiana Soto-Montero, Monica Morales-Masis

**Affiliations:** MESA+ Institute for Nanotechnology, University of Twente, Enschede 7500 AE, The Netherlands

## Abstract

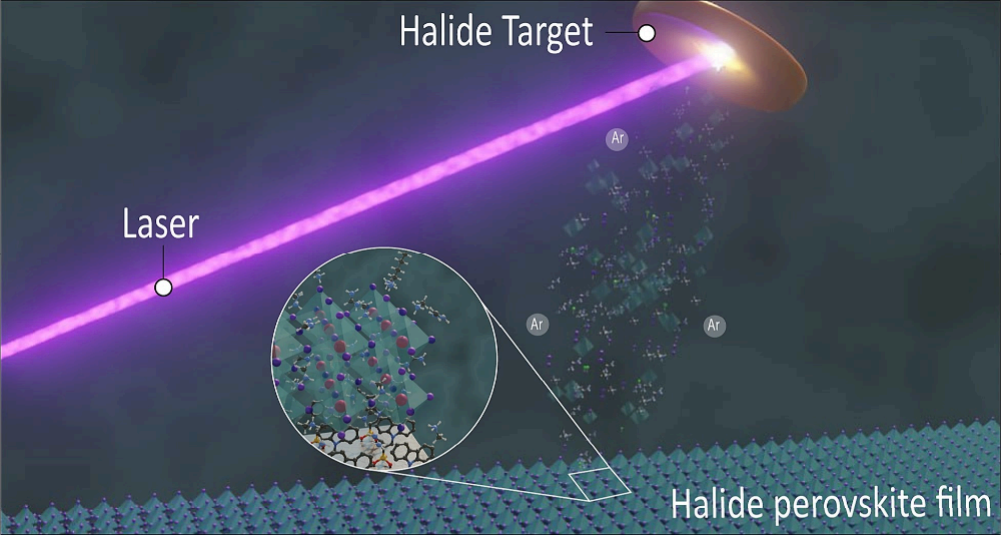

Vacuum-based or vapor-phase deposition is the most mature and widely used method for thin-film growth in the semiconductor industry. Yet, the vapor-phase growth of halide perovskites remains relatively underexplored compared to solution process deposition. The intrinsically largely distinct volatilities of organic and inorganic components in halide perovskites challenge the standard physical vapor deposition techniques. Thermal coevaporation tackles this with independent thermally controlled sources per precursor. Alternatively, pulsed laser deposition uses the energy of a laser to eject material from a target via thermal and nonthermal processes. This provides high versatility in the target composition, enabling the deposition of complex (including hybrid) thin films from a single-source target. This Perspective presents an overview of recent advances in laser-based deposition of halide perovskites, discusses advantages and challenges, and motivates the development of physical vapor deposition methods for hybrid materials, especially for applications requiring dry, conformal, and multilayer deposition.

Beyond the outstanding progress in their solar cell power conversion efficiencies (PCEs), one of the most attractive properties of metal halide perovskites (MHPs) is their opportunity for compositional tuning, allowing bandgap variations and stabilization of polymorphs via the use of cation and halide mixtures. This ability to tune the bandgap with composition has enabled significant progress in multijunction devices,^1,2^ but as the complexity in composition increases, so does the complexity in fabrication, particularly due to solubility and volatility limitations of the MHP precursors.

The widespread use of vacuum deposition techniques in industry makes vacuum-based or vapor-phase deposition of MHPs an attractive method to explore for their growth and process scalability and for future (seamless) integration of MHP deposition in photovoltaics (PV) or semiconductor production lines. Yet, MHPs fabricated via solution-based processes are leading in terms of PCE of single-junction and multijunction solar cells to date.^3,4^ There are several possible reasons for this: (1) The much larger amount of research efforts on solution processes worldwide^3^ has resulted in much faster progress on the synthesis and passivation strategies. (2) Linked to the previous point, the much lower initial investment in equipment for the solution process makes it very accessible to many laboratories. (3) Massive efforts in understanding the growth mechanisms in the solution process have resulted in materials with excellent optoelectronic quality, i.e., lower nonradiative defect density as compared to vapor-deposited perovskites.^5^

In terms of dry-processing, vacuum-based multiple-source thermal evaporation is the technique that has been able to compete in terms of technology maturity and has demonstrated performance in solar cell devices from lab-based cells to minimodules.^6−8^ In coevaporation, the perovskite absorber is deposited from two or more independent precursor sources (e.g., MAI, PbI_2_, and/or PbBr_2_) using separate temperature-controlled crucibles and controlling the deposition rate individually.^9^ This is done to decouple the distinct thermal stability and volatility of each component.^7^ The technique will, therefore, require an increasing number of sources as the complexity of composition increases, for example, when adding additives during the thin-film growth.^10−12^

Another widely used approach is the two-step deposition method, which is performed in various ways. One is a hybrid method that combines a coevaporation step for the inorganic “scaffold” (PbX_2_ or PbI_2_/CsX, with X = Cl^–^, I^–^, Br^–^) with solution postprocessing (spin coating or slot-die coating for large-area applications).^13,14^ Another approach is the sequential thermal evaporation process involving separate (alternating) coevaporation steps for the inorganic and the organic precursors followed by an annealing step.^15^ Finally, recent developments in the two-step full vapor deposition include, e.g., a combination of thermal evaporation followed by close space sublimation (CSS) of the organic counterparts.^16,17^ This results in the two-step conversion of the metal halide template into a perovskite. As a potentially scalable solution, CSS operates at distances as short as 1.4 mm between the source and the substrate, which allows for high material transfer (faster reactions) and minimal waste or decomposition of the materials. Additionally, the pressed pellet of the organic component can be used multiple times, improving the method’s throughput.^16^ Moreover, reducing the vapor transport length to a minimum appears advantageous, which is one of the main differences between thermal coevaporation (25–30 cm source-to-substrate distance) and less explored methods such as pulsed laser deposition (PLD) (5–6 cm source-to-substrate distance) or CSS (0.14 cm source-to-substrate distance). It is important to note that all sequential methods described can deliver conformal growth on textured surfaces, which is particularly interesting for, e.g., tandem Si/perovskite applications. For more details related to each specific technique, we refer readers to several review papers.^18−21^

For either coevaporation or sequential vapor deposition, the use of only thermal evaporation sources requires the independent sublimation of each precursor. Alternatively, one can use a laser source, with energy above the threshold for evaporation to, in a nonequilibrium manner, ablate a target containing all the precursors and transfer all the elements at once toward the substrate. This is the process of laser ablation, which will be elaborated below. Figure 1 represents methods relying on sublimation from thermal excitation of the source (a–c) and methods that use laser excitation of the source to deposit MHP films in a vacuum (d–f). Methods that use thermal excitation include coevaporation,^9^ flash evaporation,^22,23^ and sequential all-vapor-phase CSS.^16,17^ Methods that use laser excitation include continuous-wave (CW) laser evaporation,^24,25^ PLD,^26−28^ and resonant infrared matrix-assisted emulsion-based pulsed laser evaporation (RIR-MAPLE).^29^

**Figure 1:**
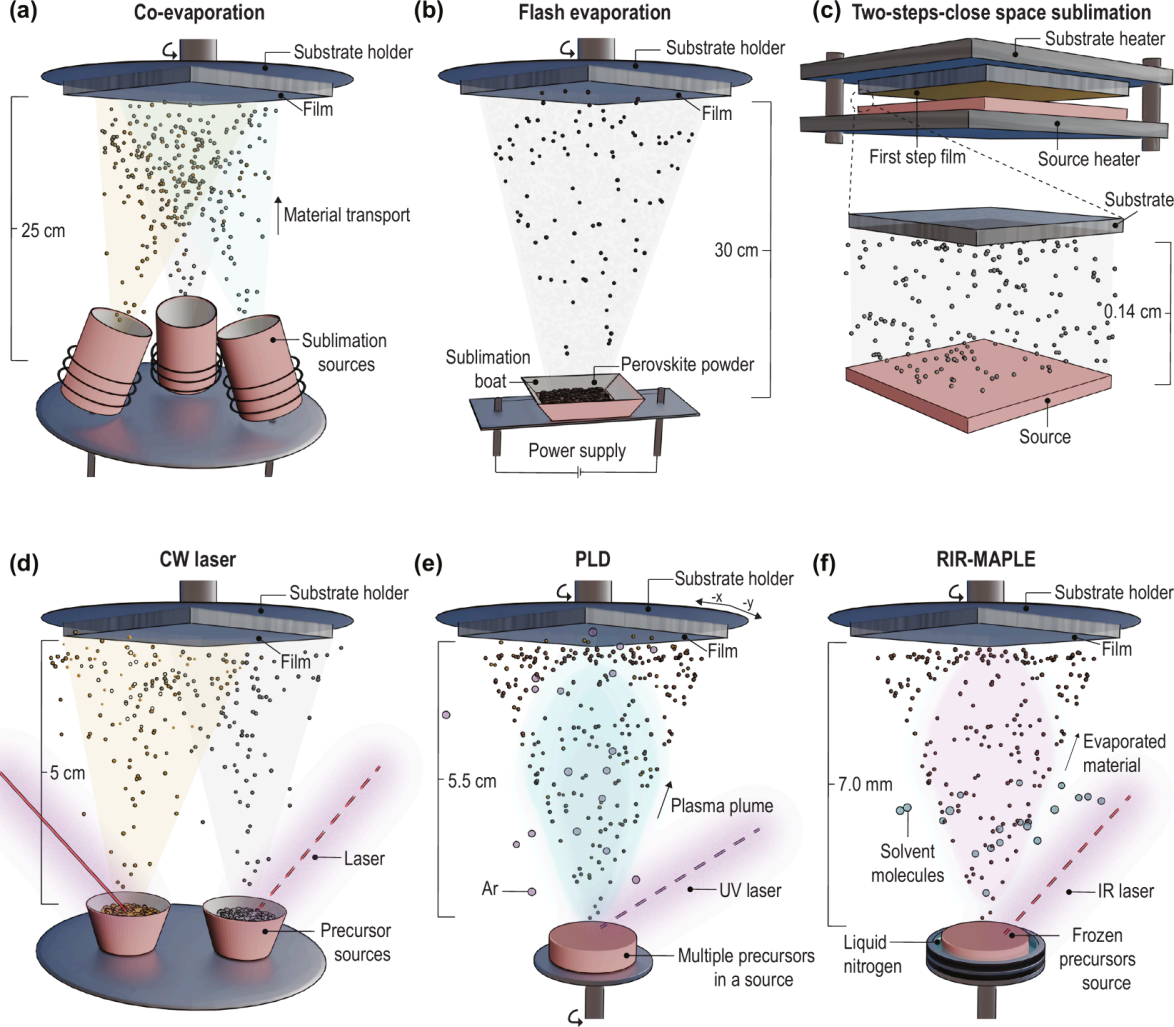
Schematics of vacuum deposition methods for vapor-phase growth of MHPs where sublimation of the source occurs via thermal excitation (a–c) or via laser excitation (d–f). The substrate-to-target distances for the different methods are also highlighted. The typical base pressures for all the methods range from 10^–5^ to 10^–7^ mbar. In terms of scalability, large-area (wafer-scale) deposition has been demonstrated for (a), (c), and (e).

## The Principle of Laser-Based Deposition and Its Interest for Single-Source Vapor Deposition of Metal Halide Perovskites

**Figure 2:**
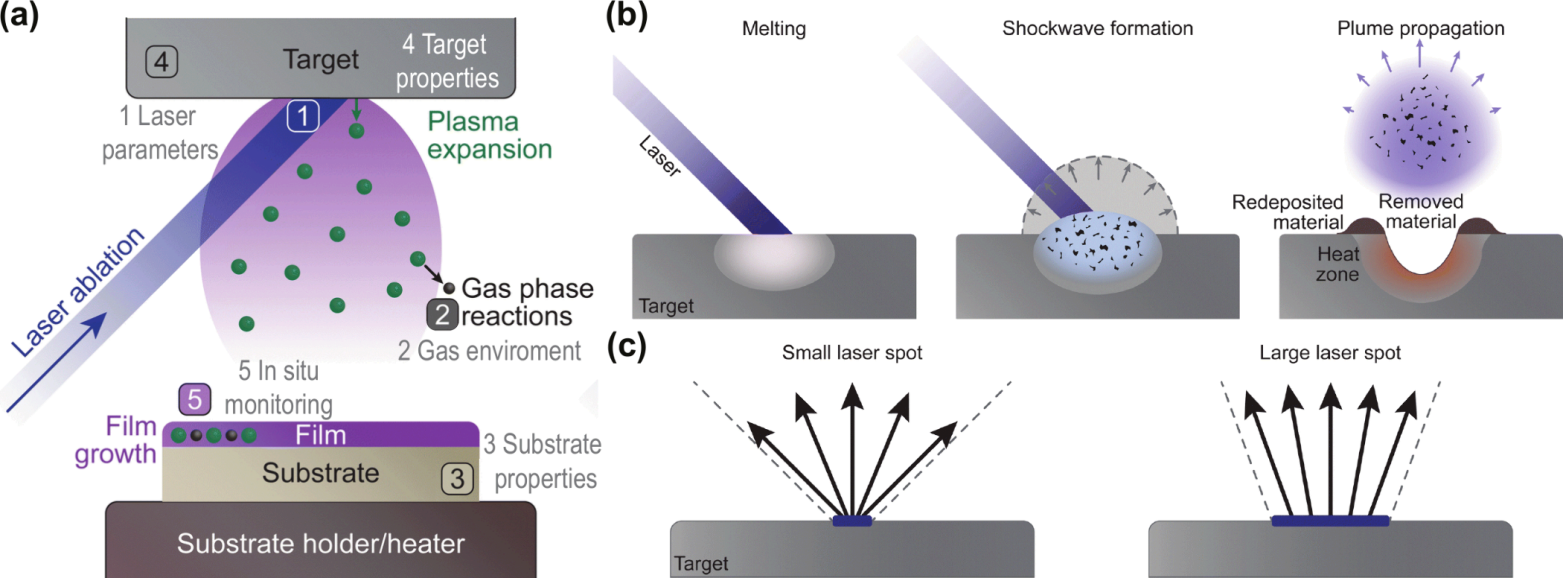
(Pulsed) laser deposition principle. (a) Overview of components and deposition parameters involved in laser deposition of thin films: 1. Laser ablation (tunable parameters: frequency, fluence, pulsed width, wavelength). 2. Gas-phase reactions (inert or reactive gas and ablated species from the target). 3. Substrate type (crystalline, amorphous, or contact layers for optoelectronic devices). 4. Target properties (define the composition and quality of the films to be deposited). 5. Thin-film growth (and possible in situ monitoring techniques to track the evolution of thin-film growth). (b) The process of laser ablation: from melting a localized area of the target to a shockwave formation to a plume propagation toward the substrate. (c) Effect of spot size on material propagation after ablation. Adapted from ref (30). Available under a CC-BY 3.0 license. Published 2024 by the Royal Society of Chemistry.

Laser-based deposition uses the principle of laser ablation. A solid target consisting of the material to be deposited is hit by a laser beam with photon energy above the bandgap of the material (if the material to ablate and deposit is a semiconductor). The laser is focused on the solid target with a defined spot size (usually in the millimeter range), and the laser fluence (energy/area) is tuned via the spot size on the source and the energy of the laser. The material transfer is initiated as the photons interact with the target material via excitation of bound electrons and subsequent transfer of their energy to the target’s material lattice through electron–phonon interactions and thermal processes. The combination of thermal and nonthermal processes during the target ablation makes the method largely volatility-insensitive, allowing the thin-film deposition of materials with complex compositions from a single source. This is undoubtedly the key advantage of PLD.^30^ In other words, the near-stoichiometric transfer of complex compounds from a single target with PLD arises from the nonequilibrium nature of the ablation process. This ablation process depends strongly on the laser fluence (i.e., an ablation threshold must be achieved). At low laser fluences, the laser pulse will heat only a small volume of the target, causing the material to evaporate through solely thermal processes. In this case, the material to be transferred will be determined by the vapor pressure of the constituents, likely resulting in nonstoichiometric transfer. By increasing the laser fluence, the ablation threshold will be reached (this is strongly material-dependent, and a preliminary ablation test is always recommended). Beyond the ablation threshold, a small volume of the target will absorb the high energy density of the laser, resulting in a nonequilibrium process that vaporizes the material independently of the vapor pressure of the constituents.^31^ In terms of MHP compositions, this means that, to deposit mixed-halide compositions and/or mixed A-side and B-side cations, the laser fluence to reach the ablation threshold must be determined (see Table S1 for typical laser fluences for various MHPs and chalcohalides). This laser fluence should not go much higher than the ablation threshold to avoid damage of the organic cations. The near-stoichiometric transfer of MHP compositions is discussed in more detail in the section “Laser-Based Deposition of Metal Halide Perovskites”. Figure 2 presents an overview of the main components and deposition parameters involved in (pulsed) laser deposition as well as the processes triggered by laser ablation of a solid target. A complete overview of PLD is found in ref (30).

The material removed from the target is transferred to the substrate (usually facing the target at a distance typically ranging from 5 to 10 cm). The laser can be pulsed (all range of frequencies possible) or continuous-wave. In the case of pulsed lasers, the material will arrive onto the substrate in a part-by-part fashion, and the frequency of the laser can be used to control the deposition rate. All the deposition parameters, i.e., deposition pressure, laser fluence, deposition rate, laser ablation spot size, substrate temperature, target properties, and target-to-substrate distance, will define the final film’s microstructure and composition. Therefore, controlling the laser ablation from a solid target with MHP precursors enables single-source vapor deposition of MHP films in one step. This simplifies the number of sources as compared to coevaporation, and with the use of good-quality targets, PLD should deliver high-quality and reproducible MHP films. However, other complexities arise, such as the laser cost and hardware for up-scaling. Directions on this will be discussed later in the text.

## Laser-Based Deposition of Metal Halide Perovskites

### Inorganic MHPs

**Figure 3:**
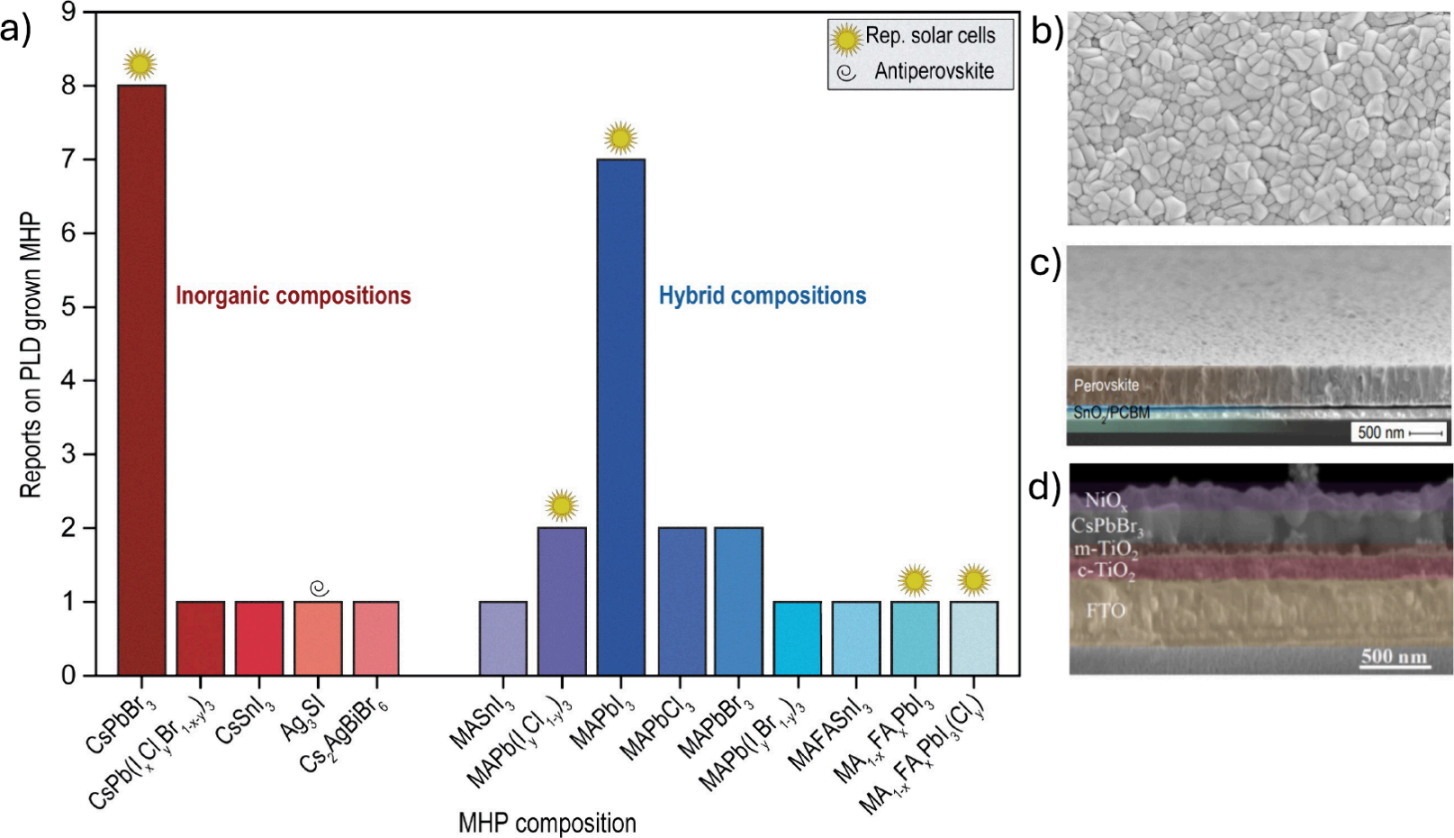
Overview of reported PLD-grown MHP films classified by compositions. CsPbBr_3_ is the most common composition reported by PLD, followed by MAPbI_3_, the archetypal hybrid MHP. Details of the growth parameters and reported devices are listed in Table S1. (b, c) SEM micrograph of PLD-grown MA_1–*x*_FA_*x*_PbI_3_ on SnO_2_/PCBM/ITO contact layers. (d) Cross-section n-i-p solar cell with PLD-grown CsPbBr_3_. Reprinted with permission from ref (34). Copyright 2023 AIP Publishing.

Among the different ways to perform laser-based depositions, PLD has been the most widely employed historically for perovskite oxides,^32^ but recently PLD has been gaining interest for energy materials such as Li-based materials for batteries^33^ and, even more recently, for MHPs for PV.^19^ Notably, for both cases, Li-based materials and MHPs, the challenge of their PVD deposition lies in the highly volatile Li^+^, I_2_, and organic species. A recent Viewpoint article has already discussed the challenges of PLD with Li^+^.^33^ In terms of MHPs, PLD of inorganic halide perovskites has been proposed in the literature (see Figure 3), and other (still few) reports to deposit hybrid halide perovskites such as methylammonium (MA^+^) and formamidinium (FA^+^) lead (or tin) iodide perovskites are emerging. The most reported inorganic perovskite deposited by PLD is CsPbBr_3_, which is not surprising due to the high stability of the material and its potential for use in light-emitting diodes (LEDs). As observed in Table S1, CsPbBr_3_ has been deposited using all ranges of laser energies (fluences) and from stoichiometric and nonstoichiometric targets. This is an indication of the robustness of CsPbBr_3_ and the feasibility to grow it by PLD for a large range of applications, such as solar cells,^34^ LEDs,^35,36^ and photodetectors,^37^ and with future prospects for integrated photonics.^38^

**Figure 4:**
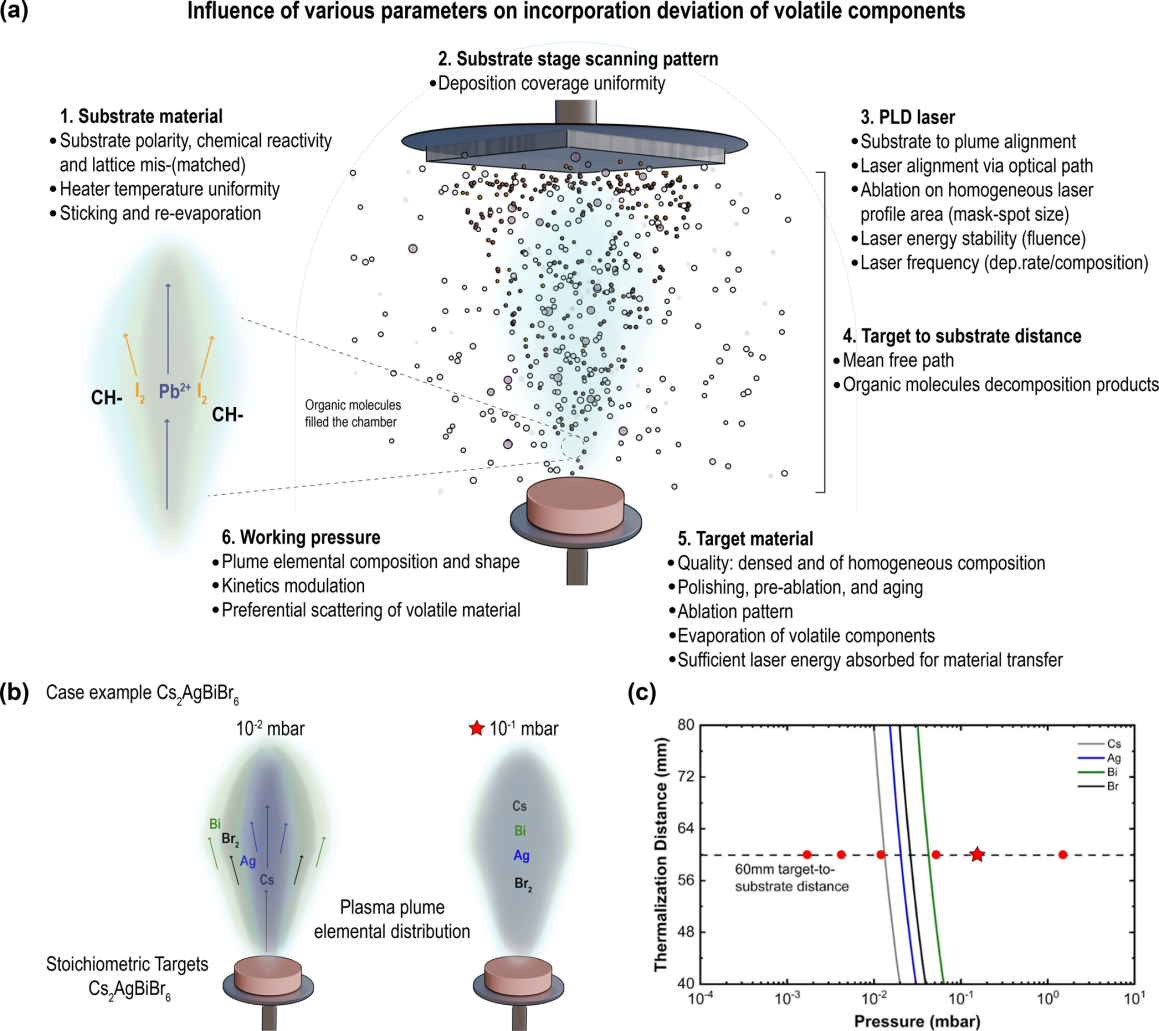
PLD process parameters that influence the composition and elemental distribution of the laser-produced plasma plume: (a) the case of hybrid MHP and (b) the case of double perovskite inorganic Cs_2_AgBiBr_6_ at two deposition pressures. (c) Thermalization distance as a function of Ar background pressure, demonstrating nearly phase-pure growth of Cs_2_AgBiBr_6_ at a target-to-substrate distance of 60 mm at 1.5 × 10^–1^ mbar (star). Reproduced from ref (27). Available under a CC-BY 4.0 license. Copyright 2021 The Authors. Published by American Chemical Society.

CsSnI_3_, a more challenging inorganic MHP, has also been successfully grown by using PLD. CsSnI_3_ presents the challenge of Sn^2+^ oxidation to the undesired Sn^4+^ state. Moreover, CsSnI_3_ presents two stable polymorphs at room temperature (RT): a photoactive (black) orthorhombic phase (γ-CsSnI_3_) and a nonphotoactive (yellow) delta phase (δ-CsSnI_3_).^39^ Yet, PLD from a stoichiometric CsI:SnI_2_ target in vacuum and while employing a low enough laser fluence (Table S1) delivered films with the photoactive orthorhombic phase, presenting a bandgap of 1.3 eV with high NIR photoluminescence (PL) emission.^28^ To avoid the oxidation of Sn^2+^, an Al_2_O_3_ capping layer was deposited in situ via PLD. Following from that work and increasing the complexity of the composition, a double perovskite, Cs_2_AgBiBr_6_, was further demonstrated by PLD.^27^ In this case, the target not only was stoichiometric but also had a double perovskite phase. This is the ultimate example of stoichiometric transfer with PLD. To achieve this, careful optimization of the deposition parameters was performed. First, the deposition pressure was optimized based on a simplified analysis of the thermalization of the species during PLD (thermalization refers to the process by which the ablated species (atoms or molecules) reach thermal equilibrium with their surroundings, in this case, the Ar gas) in relation to the target-to-substrate distance. As shown in Figure 4b, for a target-to-substrate distance of 6.0 cm, a pressure above 5 × 10^–2^ mbar is needed to thermalize all the elements of the double perovskite. Different from the MHPs described above, where I_2_ is the most vaporizable species, for the double perovskite the highly volatile species are Br_2_ and Bi^3+^. Following the Thornton model for PVD growth,^40^ the high pressures needed for thermalization of all species will lead to porous films if the deposition is done at RT. To achieve dense films, the substrate temperature was increased to enable lateral diffusion of species in the substrate during growth. A temperature of 200 °C was found to be sufficient to achieve large grains while avoiding re-evaporation of Br_2_ or Bi^3+^ from the film (Figure 4c).

Beyond MHPs, the fabrication of the chalcohalide antiperovskite Ag_3_SI was enabled by thermal-assisted mechanochemical synthesis followed by PLD to demonstrate and characterize the material in thin-film form.^41^ Interestingly, this report marked (up to the date of this publication) the lowest temperature and shortest time reported to synthesize Ag_3_SI. To achieve films with the stoichiometric ratios for the Ag_3_SI antiperovskite phase, two strategies were used: (1) The PLD target contained an excess of elemental sulfur (S), with a 3:1 molar ratio of S:Ag_3_SI (mechanosynthesized). (2) The films were postannealed at 200 °C for at least 30 min in a N_2_ atmosphere to remove any excess sulfur, resulting in films with a 3:1:1 Ag:S:I composition. More details on the material properties are available in ref (41).

### Hybrid MHPs

**Figure 5:**
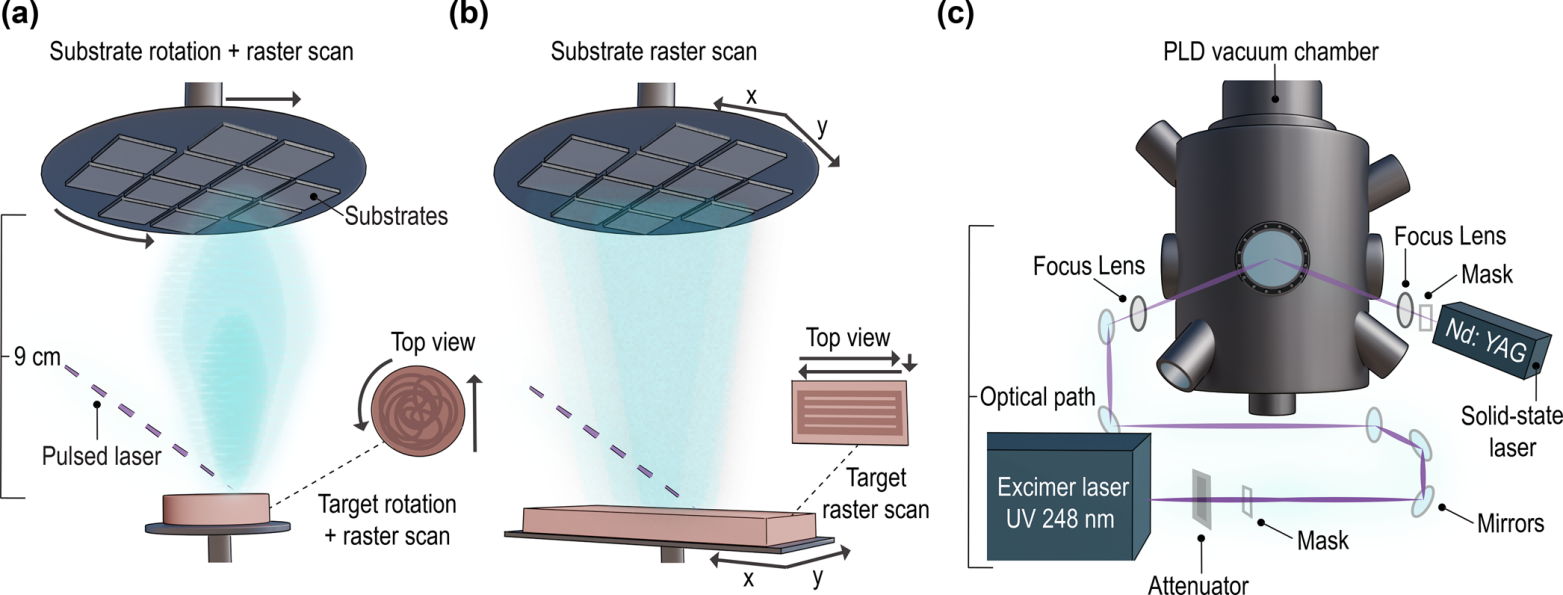
Wafer-scale PLD with two examples of substrate and target scanning to achieve large-area coating: (a) substrate rotation and raster scan + target rotation and raster scan and (b) substrate raster scan in X-Y directions and target raster scan with an optimized ablation pattern on the target. (c) Comparison of the different optical paths needed for PLD with an excimer laser and for (P)LD with a solid-state laser. Adapted from ref (60). Available under a CC-BY 4.0 license. Copyright 2023 The Authors. Published by Springer Nature.

Regarding hybrid MHPs, the first reports were from Bansode et al. on the fabrication of hybrid perovskite films using off-axis PLD.^42^ The process was done with a single target and a UV excimer laser (KrF, 248 nm) in an off-axis geometry. In this geometry, the substrate is parallel to the plume and not perpendicular (as typically done in an on-axis configuration), which was done to prevent the escape of highly vaporizable atoms such as iodine (I_2_) and the organic components. The solid target composition was PbX_2_ (X = I^–^, Cl^–^, or F^–^) and MAI homogeneously distributed in a solid pressed target. While this technique is an interesting approach, it presents the disadvantage of low reproducibility, as the film composition will be extremely sensitive to the substrate position with respect to the edge of the plume, likely affecting the growth control as well. To overcome the challenges of high volatility of the organic species and I_2_, while still using the standard on-axis configuration, i.e., target facing the substrate, an excess of MAI (and FAI) can be used in the target. In a systematic study of PLD of MAPbI_3_, a MAI:PbI_2_ ratio of 8:1 in the target was demonstrated as optimum for the fabrication of MAPbI_3_ films in a single step at RT (no postannealing needed). A follow-up study showed that this ratio of organic:inorganic = 8:1 also holds for the PLD-grown MA_1–*x*_FA_*x*_PbI_3_ perovskites. Importantly, it was also demonstrated that the MA^+^: FA^+^ ratio was maintained from target to film when the PLD was conducted in stationary mode, i.e., target constantly facing the substrate, which is the most common way PLD was historically executed for perovskite oxides but presents the disadvantage of limited deposition area. For large-area depositions, target scanning and wafer rotation or scanning are required,^43,26^ where the plasma plume will locally but dynamically coat the substrate over a full area. The substrate scanning speed and scanning pattern also need to be optimized to ensure full area coverage with a uniform composition and thickness (Figure 5). When PLD in scanning mode is used, the ratio MA^+^:FA^+^ from the target to film differs,^44^ which is related to the highly dynamic processes occurring, where not only the rate of arrival of the species plays a role but also the sticking of the molecules on the substrate. However, once the target and film composition are determined (calibrated), the process is highly reproducible. It is important to mention here that scanning PLD will be the way to go for process scalability, and therefore, understanding the processes occurring during the material transfer from target to substrate is of utmost importance (more on this in the following section).

The understanding and control of the MA_1–*x*_FA_*x*_PbI_3_ growth with scanning PLD allowed us to take further steps. Inspired by Lohmann et al.’s work on the addition of PbCl_2_ in coevaporated FA_1–*y*_Cs_*y*_Pb(I_*x*_Cl_1–*x*_)_3_,^10^ we first added PbCl_2_ (20 mol% with respect to PbI_2_) as the fourth precursor to the MAI + FAI + PbI_2_ target. This was done with the goal of achieving bulk passivation during the one-step PLD growth and to demonstrate efficient solar cells with PLD-grown MA_1–*x*_FA_*x*_Pb(I_*y*_Cl_1–*y*_)_3_.^45^ p-i-n single-junction devices with a PCE of 15.5% were demonstrated for as-grown PLD-MA_*y*_FA_1–*y*_Pb(I_*x*_Cl_1–*x*_)_3_. When the surface of the PLD-MA_*y*_FA_1–*y*_Pb(I_*x*_Cl_1–*x*_)_3_ was passivated with a solvent-based OAmI post-treatment, a boost in open-circuit voltage (*V*_oc_) from 995 mV to 1137.7 mV led to PCEs of 19.7%.^45^ This is, to our knowledge, the highest PCE for PLD-based perovskite solar cells and validates the potential of this single-source vapor deposition technique for PV application. Notably, there is a lot of room for optimization regarding cell structure, passivation, compositions (e.g., for wide- and narrow-bandgap perovskites), and overall PCEs, which are expected to happen as the research community studying PLD of MHPs grows and efforts intensify on device optimization.

In addition to the excimer UV lasers, other lasers have also been used for MHP laser deposition. For example, in AIST-Japan, two 808 nm CW semiconductor lasers were used to irradiate a CH_3_NH_3_I (MAI) and PbI_2_ powder target individually to co-deposit the MAPbI_3_ perovskite.^24^ However, MAI has no absorption of 808 nm and required silicon-powder assistance to absorb the laser energy. This approach, however, was recently optimized by employing a 405 nm CW laser on a single crystal of MAPbBr_3_, demonstrating stoichiometric transfer from target to thin film.^25^ Another approach was taken at Duke University, where RIR-MAPLE^31^ was used to demonstrate MAPbI_3_ solar cells with 12.2% PCE.^46^ The MAPbI_3_ films were deposited from a single target made of an ethylene glycol (MEG)-based matrix embedding an emulsion composed of PbI_2_ and MAI mixed with DMSO as the solvent. Using a Q-switched Er:YAG laser (2.94 μm), the frozen target containing the mixed solution was targeted, forming a hybrid plume that directly transferred the material to the substrate. Following the principle of RIR-MAPLE, the laser was absorbed by the MEG matrix and therefore did not damage the MAPbI_3_ source.^46,47^ This method combines a solution-based target with deposition in-vacuum, allowing for, e.g., composition and thickness control (in a similar matter to PLD and other vapor deposition techniques). Yet, the complexity of the method (e.g., the cryogenically cooled target), including selectively pumping out the solvents, limits the versatility and scalability of the method.

Table S1 presents an overview of reported MHP deposited by PLD (and other laser sources beyond pulsed excimer lasers) with a list of the common growth parameters used and whether devices were fabricated. Notably, PLD of MHPs is an emerging topic, and a very limited number of solar cells have been reported. Figure 3 categorizes the number of reports per PLD-grown MHP composition.

As mentioned earlier, one of the key properties of PLD is the ability to enable near-stoichiometric transfer from a single-source target of complex compositions to a thin film. But to achieve this, the composition and elemental distribution of the laser-produced plume need to be controlled. Figure 4a and 4b illustrate the possible elemental distribution of the plasma plume species after ablation of a hybrid MA^+^- or FA^+^-containing target and an inorganic Cs_2_AgBiBr_6_ target. Heavy elements such as Pb^2+^ are confined in the center of the plume (low scattering probability), while lighter elements such as I_2_ and organic molecules have higher scattering probability and therefore form a more extended plume. While this is yet to be demonstrated experimentally for PLD-grown MHPs, the scattering probability of light elements in PLD has been extensively studied and demonstrated for Li-based or other oxide materials.^30,33,48^ One strategy applied for the Cs_2_AgBiBr_6_ target (as commented in the text above) is to increase the deposition pressure (Ar pressure) to thermalize the species in the plasma and achieve stoichiometric deposition.^27^ For the hybrid MHPs, the highly volatile organic cations, in several cases added in excess in the target, are hypothesized to likely fill the chamber. This creates an atmosphere with a mixture of Ar, organic halide precursors, and likely also subproducts, following similar reports of coevaporation.^49,50^

Yet we note that the growth of stoichiometric MAPbI_3_ or MA_1–*x*_FA_*x*_PbI_3_ using a PLD target that is highly enriched with the organic molecules (8-fold) causes an overpressure of the volatile species in the gas phase that seemed to self-regulate at the growth front of the thin film, forming the ABX_3_ compound.^26,45,51^ The initial stages of the growth, i.e., nuclei formation and growth, are strongly dependent on substrate type. In a recent work from our group, it was shown that, using equal PLD parameters to grow MAPbI_3_ films, the growth on crystalline lattice-matched substrates is different from the growth on amorphous substrates. Growth of MAPbI_3_ on freshly cleaved lattice-matched KCl substrates results in a direct epitaxial relation between the substrate and film. Moreover, the MAPbI_3_ phase forms from the initial growth stages.^52^ Contrary to this, growth on amorphous substrates or contact layers for solar cells results in the formation of a PbI_2_-rich layer up until ∼20 nm which slowly converts to MAPbI_3_ or MA_1–*x*_FA_*x*_PbI_3_ as MAI and FAI react with the PbI_2_-rich nuclei.^44^ Note that the type of growth and the dependence on substrate type resemble those of the growth mechanism also reported for coevaporation.^53^ This indicates that, while the sublimation sources are different for coevaporation vs laser ablation, one being multisource and the other one single-source, the vapor-phase growth follows a general mechanism when the species condense onto the substrate, with the substrate type influencing the morphology, composition, film orientation, and overall type of growth.

Besides laser ablation for vapor-phase growth of MHPs, laser ablation has also been used to post-treat the surface of MHPs.^54^ An UV pulsed laser (λ = 355 nm) is used to carefully polish the surface of the MHP film, achieved via careful control of the laser energy and scanning speed. The authors demonstrated an improvement in the *V*_oc_ of the perovskite solar cells after laser polishing, indicating the removal of defects or uneven compositions at the surface. This is just one example of the versatility of using lasers as solvent-free strategies to grow thin films but also modify (or treat) the surface of films. An understanding of the laser parameters such as laser fluence, frequency, and material absorption properties is required to achieve high tunability, controlled light–matter interactions, and, from there, novel functionalities for material growth, surface post-treatments, and likely even phase modifications/transitions. Laser patterning (scribing), laser annealing, and laser transfer are, as well, just a few other applications of laser-based processes.

## Perspective: Light to Matter, Matter to Light

(Pulsed) laser deposition of MHPs is emerging as an alternative vapor-phase deposition method for the synthesis of MHPs. The advantages of PLD, as listed previously in this Perspective, are the possibilities to use several times a single-source target that contains all the perovskite precursors and, via controlled laser ablation, deposit thin-film materials of complex compositions. PLD, however, is an expensive method, mainly due to the high cost of the traditionally used high-energy UV excimer lasers. Below we describe some of the developments in the field toward scalability of the technique, challenges, and opportunities. (A summary of the discussed advantages, challenges, and opportunities of PLD of MHPs is presented in Table S2.)

### Wafer-Scale PLD

One of the first developments in the PLD field toward scalability was the demonstration of wafer-scale PLD.^31^ Wafer-scale PLD allows for the uniform deposition of films over large substrates and has been scaled to the typical wafer sizes used in semiconductor fabrication. Achieving uniform film thickness and composition across the entire wafer surface is crucial. Therefore, PLD systems need to be designed to ensure precise control over target and substrate scanning parameters (Figure 5a,b). Wafer-scale PLD systems are commercially available, and they are increasingly being used for a variety of materials and applications, including low-damage transparent conductive oxide (TCO) deposition for solar cells (see the note in the SI about the difference between PLD of TCOs and MHPs).^43,55−57^ Interestingly, a breakthrough on wafer-scale PLD development occurred this year when LAM Research introduced the world’s first production-oriented PLD tool.^58^ While the development is specifically for next-generation piezo-based radio frequency (RF) filters, it represents the scalability potential for other applications requiring complex thin-film deposition.

### Deposition Throughput

Compared with other PVD methods, PLD generally has lower throughput. Currently, techniques such as sputtering or evaporation can achieve higher deposition rates suitable for industrial-scale production, while PLD is more suitable for applications requiring precise control over film composition and microstructure. Yet, developments in laser technology, system automation, and process optimization are expected to continue to improve the throughput of PLD. Moreover, the fact that several of the MHP applications require RT deposition and polycrystalline films facilitates the up-scaling and throughput of the process as compared to the use of PLD for high-temperature perovskite oxide films. Cost, throughput, and deposition rate will be decisive criteria to consider PLD or any other form of laser-based deposition for the fabrication of MHP for PV^3^ or for more specialized optoelectronic applications such integrated photonics, superlattices for efficient LEDs, and more.^59^

### Alternative Laser Sources

High-energy UV excimer lasers, typically KrF (248 nm), present ideal characteristics for PLD, including their high photon energy, short wavelength, efficiency, and clean ablation process related to high beam stability. UV excimer lasers have short pulse durations (typically in the nanosecond range), which means the energy is delivered in very short bursts. This reduces the heat-affected zone on the target (Figure 2b), minimizing thermal damage, which is especially beneficial for MHP targets. Yet, excimer lasers require high investment and maintenance costs, making their use less attractive for technologies such as PV. However, laser-based deposition of MHPs could be achieved by using lower cost lasers. Alternatives include solid-state Nd:Y_3_Al_5_O_12_ (Nd:YAG) lasers, which possess a light and compact design, have tunable wavelengths, e.g., 532 or 355 nm, with frequency-doubling crystals, can be pulsed, and require laser paths as short as 20 cm (Figure 5c).^60^ Recent advances in Nd:YAG lasers have demonstrated uniformity in the overall laser beam profile and high stability in laser energy.^60^ Finally, CW lasers as demonstrated in ref (25) represent a low-cost alternative but remain to be demonstrated for MHP film thicknesses above 100 nm, including analysis of the target stability for long deposition times.

### Availability of Commercial Targets

While there is a multitude of companies offering PVD targets for oxide materials and metals, the availability of halide-based targets for PVD is still limited (or nonexistent), and most of the research still relies on in-house-developed targets and processes. This limits the comparability of results between laboratories and can lead to large variation in the quality of the reported laser-deposited MHP films. Considering the significant role that the target plays in the thin-film quality, the availability of high-quality targets would benefit the further development of laser-based deposition of MHPs.

In conclusion, laser-based deposition is a promising fabrication method for the vapor-phase growth of MHPs in thin films. With several MHP compositions reported and promising optoelectronic devices developed with PLD-grown MHPs, the potential of this method is demonstrated. Yet, there is plenty of room for research innovations on PLD or other laser-based methods to grow MHP films either for established applications such as PV, including tandem devices, or for more specialized applications such as (epitaxial) heterostructures for LEDs or integrated photonics. Finally, as our understanding of laser ablation of halide perovskite targets progresses, simpler, cheaper, and up-scalable laser deposition processes can be developed. This furthermore motivates the development and exploration of physical vapor deposition methods for the fabrication of MHP films, where conformality and dry and additive processing are required.

## Supporting Information Available

Table S1, overview of key growth parameters of reported MHPs using laser-based deposition techniques, corresponding thin-film properties, and reported optoelectronic device characteristics; Table S2, summary of advantages, challenges, outlook and opportunities for future developments of PLD of MHPs; comment about the importance of reporting key PLD deposition parameters; and comment about the difference between PLD of TCOs and PLD of MHPs (PDF)
