# Supplementary material for: Laser Deposition of Metal Halide Perovskites
Source: ACS Energy Lett. 2024 Aug 1;9(8):4199–208. doi: 10.1021/acsenergylett.4c01466 (PMC11320642; doi:10.1021/acsenergylett.4c01466)
Supplement: Supplementary file 1 — nz4c01466_si_001.pdf [file nz4c01466_si_001.pdf]

Supplementary Information

Laser Deposition of Metal Halide Perovskites

Tatiana Soto-Montero[1], Monica Morales-Masis[1,]*

[1]MESA+ Institute for Nanotechnology, University of Twente, Enschede, 7500 AE, The Netherlands.

AUTHOR INFORMATION

Corresponding Author

*m.moralesmasis@utwente.nl

Table S1. Overview of key parameters influencing thin film growth of metal halide perovskites using laser-based deposition techniques, resulting thin film properties, and reported optoelectronic device characteristics.

MHP composition	Target composition	(B or D)* Pressure (mbar) / #pulses	Laser type / frequency (Hz)	Laser energy (mJ) / fluence ($J\,cm^{-2}$)	Substrate temp (°C) / T-S distance (cm)	Growth rate nm s^{-1}/ film thickness nm	Post ann. temp (°C) /conditions	Substrate type, optoelectronic device	Device notes	Ref
$CsPbBr_3$	CsBr: $PbBr_2$, 1: 1	(B) $1x10^{-6}$ / 6000	KrF 248 nm / 5	300 / NA	RT / 7.5	NA	150 / 1.0 mbar N_2 atmosphere for 1h	Sapphire (2 x 1 cm), towards on-chip photonics	NA	11
$CsPbBr_3$	CsBr: $PbBr_2$, 1: 1	(B) < $1x10^{-6}$ / NA	KrF 248 nm / 5	NA	120 / NA	0.1 / 100	150 / during the in-situ deposition of ZnO	p-GaN, single-chip GaN-based white LEDs	NA	12
$CsPbBr_3$	Single crystal powders by ITC	(B) < $3x10^{-5}$ / 200-600	KrF 248 nm / 5	100/ NA	320 / 5.0	NA / 100-290	350 / for 2h in the chamber	FTO/c-TiO_2/m-TiO_2, Solar cells	Aver.5.3%, Max. 6.32% PCE, no hysteresis or stability data reported	13
$CsPbBr_3$	CsBr: $PbBr_2$, 1: 2, ITC method	(B) < $5x10^{-6}$ / 400	KrF 248 nm / 5	100 / 0.30	350 / 5.0	2.5 / 200	350 / for 1h	FTO/c-TiO_2/m-TiO_2, Solar cells	5.47% PCE, unencapsulated retained 95% PCE for 1200 h at 85 °C and 30 RH.	14
$CsPbBr_3$	CsBr: $PbBr_2$, 1: 6.7 (and 1: 1), post-ann. 300-550 °C for 2h	(B) < $5x10^{-7}$ / 2000	KrF 248 nm / 5	NA / 2.0 and 5.0	RT / 4.0	Growth analysis / 365-530	NA	Silica, NA	NA	15
$CsPbBr_3$	CsBr: $PbBr_2$, 1: 1.2 (and 1: 1), post-ann. 547 °C for 2h	(B) $1.3x10^{-7}$ / NA	KrF 248 nm / 1	NA / 1.0	317 / NA	NA / 60	NA	4H-SiC, position-sensitive detector (PSD)	PSD: up to 827 mV/mm	16
$CsPbBr_3$	CsBr: $PbBr_2$, 1: 1	(B) $1.x10^{-8}$ / NA	KrF 248 nm / 5	250 / NA	From 20-200 / NA	1.5-1.8 / 3000	NA	Si (100), photodetector	peak responsivity: 0.6 A/W and detectivity: 9.5 × 10^{11} $cmHz^{1/2}/W$ (520 nm, −5V)	17
$CsPbBr_3$	CsBr: $PbBr_2$, 1: 1, post-ann. 400 °C for 2h + 500 °C for 2h.	(D) $2.0x10^{-4}$ -10^{-2} / 1200-4500	KrF 248 nm / 2-10	NA / 1.0	RT / 3-6	NA / 120	NA	Silica, NA	NA	18

*B: background pressure, D: deposition pressure, post.ann.: post-annealing, CW: continuous wave laser.

MHP composition	Target composition	(B or D)* Pressure (mbar) / #pulses	Laser type / frequency (Hz)	Laser energy (mJ) / fluence (J cm^{-2})	Substrate temp (°C) / T-S distance (cm)	Growth rate nm s^{-1}/ film thickness nm	Post ann. temp (°C) /conditions	Substrate type, optoelectronic device	Device notes	Ref
$CsPb(I_xCl_yBr_{1-x-y})_3$	PbX_2: CsX (X=Cl/Br/I) 1:1, post-ann. 300 °C for 12 hours	(D) $1.0x10^{-3}$ / 300	KrF 248 nm / 5	NA / 0.5	NA /4.0	NA / 120	200 and 450	(001)-$SrTiO_3$(STO) / NA	NA	19
$CsSnI_3$	CsI: SnI_2, 1: 1	(D) $1x10^{-3}$ / 4000	KrF 248 nm / 5	NA / 0.2	RT / 5.0	0.25 / 200	NA	Si (with native SiOx), fused silica, and glass substrates	NA	20
Ag_3SI	Ag_3SI: S-enriched, 1: 3 post-ann. 160 °C for 30 min (inert atmosphere)	(D) $1.5x10^{-2}$ / 12000	KrF 248 nm / 10	NA/ 0.75	RT / NA	NA / 140	200/ for 30min in a N_2 atmosphere	glass, ITO/SnO_2 (3 x 3 cm)	NA	21
$Cs_2AgBiBr_6$	CsBr: AgBr: $BiBr_3$, 1: 1: 1	(D) $1.5x10^{-1}$ / 12000	KrF 248 nm / 8	NA/ 0.45	200 / 6.0	0.07 / 300	annealing causes Ag(s) formation	Si (with native SiOx) and fused silica	NA	22
$MASnI_3$	SnI_2: MAI (1: 4, off-axis)	(B) $1.3x10^{-7}$ / NA	KrF 248 nm / 5	NA / 0.3	RT / NA	NA	NA	Glass (2 x 2 cm), NA	NA	23
$MAPb(I_yCl_{1-y})_3$	PbI_2: MAI, 1: 12, post-ann. 100 °C for 1 h in the glovebox	(D) 0.13, gas mixture Ar:H_2 (90%:10 %)/NA	KrF 248 nm / 5	NA / 0.3	100 / 5.0	0.6 / 500	NA	CuO_2 and TiO_2/ photosensor and solar cells	fast photo response rise time τrise > 0.2 s / 10.9% PCE	24
$MAPb(I_yCl_{1-y})_3$	$PbCl_2$: MACl :MAI, 1: 2: 2	(B) $1.3x10^{-7}$ / NA	KrF 248 nm / 5	NA / 0.3	RT / NA	0.19 / 500	as deposited RT	ITO/ZnO, Solar cells	7.7% PCE, observed hysteresis	23
$MAPbI_3$	PbI_2: MAI, 1: 18 on-axis (1: 4, off-axis)	(B) $1.3x10^{-7}$ / NA	KrF 248 nm / 5	NA / 0.3	90 (on axis), RT (off axis) / NA	NA	NA	Glass (2 x 2 cm), NA	NA	23

MHP composition	Target composition	(B or D)* Pressure (mbar) / #pulses	Laser type / frequency (Hz)	Laser energy (mJ) / fluence ($J\ cm^{-2}$)	Substrate temp (°C) / T-S distance (cm)	Growth rate $nm\ s^{-1}$/ film thickness nm	Post ann. temp (°C) /conditions	Substrate type, optoelectronic device	Device notes	Ref
$MAPbI_3$	PbI_2: MAI, 1: 8	(D) ≈10^{-2}/ NA	KrF 248 nm / 4	NA / 0.3	RT / 6.0	NA / 200-500	as deposited RT	Si (with native SiOx), fused silica, ITO/SnO_2/PCBM	NA	[25]
$MAPbI_3$	PbI_2: MAI, 1: 1, DMSO/MEG (cryogenically frozen solvent matrix 0.022 M)	(D) $4.0x10^{-4}$ / NA	Er: YAG 2.94 µm/ 2	NA / 0.125-0.135	10 / 7.0	≈ 0.02 / ~230 to ~340	110 / for 10 min in a nitrogen-filled glovebox	FTO/NiOx, solar cells	12.2 % stabilized PCE, observed hysteresis	[26]
$MAPbI_3$	PbI_2: MAI, 1: 8	(D) $3.0x10^{-2}$ / 6000, 3000, 1500	KrF 248 nm / 1	NA / 0.32	RT / 5.5	NA / 70, 40, 13	as deposited RT	KCl (100)	NA	[27]
$MAPbI_3$	PbI_2: MAI, 1: 3	(D) 1.0 / 100	Nd: YAG 532 nm / NA	NA / 2.5	RT / 5.0	NA / 450	as deposited RT	Si (111), n-Si photodetector	maximum responsivity of 2.57 A/W	[28]
$MAPbI_3$	PbI_2: MAI, 1: 1, DMSO/MEG (cryogenically frozen solvent matrix 20 mM)	(D) $4.0x10^{-4}$ / NA	Er: YAG 2.94 µm/ 2	NA / 0.125-0.135	25 / 7.0	NA	NA	Soda-lime glass	NA	[29]
$MAPbI_3$	PbI_2: MAI, 1: 10	(B) $4x10^{-6}$ / 12000	KrF 248 nm / 5	NA/ 0.4	RT / 5.0	0.4 / 600	NA	glass and n-Si, photodetector	rise and fall time, 58.8 µs and 1.9 µs respectively	[30]
$MAPbI_3$	MAI + silicon powder and PbI_2 powder sources for codeposition	(D) $1x10^{-5}$/ NA	CW, 808 nm/ 10 square wave	NA	NA	0.045/ 200	NA	PEDOT:PSS/PCDTBT; NiOx/PCDTBT, solar cells	15.6% average, 16% best cell	[31]
$MAPbCl_3$	$PbCl_2$: MACl, 1: 1, DMSO/MEG (cryogenically frozen solvent matrix 20 mM)	(D) $4.0x10^{-4}$ / NA	Er: YAG 2.94 µm/ 2	NA / 0.125-0.135	25 / 7.0	NA	NA	Quartz	NA	[29]

MHP composition	Target composition	(B or D)* Pressure (mbar) / #pulses	Laser type / frequency (Hz)	Laser energy (mJ) / fluence ($J\ cm^{-2}$)	Substrate temp (°C) / T-S distance (cm)	Growth rate nm s^{-1}/ film thickness nm	Post ann. temp (°C) /conditions	Substrate type, optoelectronic device	Device notes	Ref
$MAPbCl_3$	$PbCl_2$: MACl 1: 4 off-axis	(B) $1.3x10^{-7}$ / NA	KrF 248 nm / 5	NA / 0.3	RT / NA	NA	NA	Glass (2 x 2 cm), NA	NA	23
$MAPbBr_3$	$PbBr_2$: MABr, 1: 1, DMSO/MEG (cryogenically frozen solvent matrix 20 mM)	(D) $4.0x10^{-4}$ / NA	Er: YAG 2.94 µm/ 2	NA / 0.125-0.135	25 / 7.0	NA	NA	Soda-lime glass	NA	29
$MAPbBr_3$	$PbBr_2$: MABr, 1: 12 (on-axis), post-ann. 100 °C for 1 h in the glovebox	(D) 0.13 gas mixture Ar:H_2 (90%:10 %)/NA	KrF 248 nm / 5	NA / 0.3	100 / 5.0	0.4 / NA	NA	NA	NA	24
$MAPbBr_3$	$PbBr_2$: MABr, 1: 1 single crystals or pellets	(D) $1x10^{-2}$–$3x10^{-2}$	CW, 405 nm /NA	NA/ ≈2.0	25/ 5.0	0.055/100	as deposited RT	FTO, glass, Si(with native SiOx),	NA	32
$MAPb(I_yBr_{1-y})_3$	($PbBr_2$ + PbI_2) : (MAI + MABr), 1: 12	(D) 0.13 gas mixture Ar:H_2 (90%:10 %)/NA	KrF 248 nm / 5	NA / 0.3	100 / 5.0	NA	NA	NA	NA	24
$MAFASnI_3$	SnI_2: (0.2 · MAI + 0.8 · FAI), 1: 8 post-ann. 160 °C for 2.5h	(B) $1.5x10^{-6}$ / NA	KrF 248 nm / 5	80-200/ 0.16-0.41	20-120/ NA	NA / 250-975	below 60	silicon (911)	NA	33
$MA_{1-x}FA_xPbI_3$	PbI_2: (0.75 · MAI + 0.25 · FAI), 1: 8	(D) 3.0-$2.0x10^{-2}$ / 12000 or 13500	KrF 248 nm / 4	NA / 0.31	RT / 5.5	NA / 500	as deposited RT	ITO/SnO_2/PCBM (3 x 3 cm), solar cells	14.0% PCE, observed hysteresis	2
$MA_{1-x}FA_xPbI_3(Cl_y)$	(0.8 · PbI_2 + 0.2 · $PbCl_2$) : (0.75 · MAI + 0.25 · FAI), 1: 8	(D) $2.0x10^{-2}$ / 18000	KrF 248 nm / 4	NA / 0.31	40 / 5.5	0.1 / 440-400	100 / 10 min N_2 atmosphere	ITO/2PACz (2.5 x 2.5 cm), solar cells	15.6% PCE and 19.7% PCE without/with 2D passivation, thermal stability test	9

On the importance of reporting key deposition parameters.

To ensure the reproducibility of thin film growth, detailed experimental information is essential. In the context of pulsed laser deposition (PLD), critical deposition parameters include: 1) fluence (energy/area J/cm^2), 2) deposition or working pressure (and gas(es) employed), 3) spot size on the target (mm^2), 4) target composition, 5) target-to-substrate distance, 6) laser frequency, and 7) heater temperature. These parameters are crucial for replicating across different laboratories or PLD vacuum systems. However, as noted in Table S1, many studies fail to provide comprehensive details on these deposition parameters, hindering progress in the field. Beyond properly reporting deposition parameters, other factors such as target preparation, raster and/or scanning patterns on target or of the heater stage, substrate or device stack type, and chamber shape and volume can also impact the reproducibility of results between PLD chambers, even for the same user. Therefore, while reporting deposition parameters should be seen as a guideline, minor modifications are always expected.

PLD of TCOs vs. PLD of MHPs.

The difference between PLD of TCOs and PLD of MHPs comes down to the intrinsic properties of the materials. TCOs (and several metal oxides in general) have higher formation energies than halide perovskites. Therefore, the laser fluence required for target ablation is higher for oxides than for halide perovskites. Specifically, a typical laser fluence used for TCOs is ~2 J/cm^2,[1] and for halide, perovskites is one order of magnitude lower, i.e., 0.2-0.3 J/cm^2.[2]

The UV excimer laser works for both materials because it has an energy greater than the band gap of both (ensuring absorption of the photons). For hardware and process optimization, the requirements are the same for both materials. Both can be deposited at room temperature, but TCOs will be mainly amorphous, while MHPs could already be crystalline as deposited. This difference in crystallinity is again correlated to the low formation energy of the MHPs.

Another difference is the availability of targets. TCO targets are widely commercially available, but halide perovskite targets are not commonly available. As a result, research currently relies on in-house-made targets for MHPs.

Table S2. Summary of advantages, challenges, outlook, and opportunities for future developments of PLD of MHPs.

	Advantages	Challenges	Outlook and Opportunities
Composition tunability	Complex compositions are transferred from a single source target to the film.[3]	Precursor ratio must be tuned in the target based on deposition conditions to obtain the desired stoichiometry in the films.[4]	In-situ monitoring of plasma composition and dynamics to understand and predict material transfer.[5]
Scalability	Wafer-scale PLD is available.<br>First industrial PLD demonstrated.[6]	Laser maintenance cost.[7]<br>Complex raster scanning patterns of substrate and targets to deliver uniform thin films.[8]	Large (> 4 inch) halide targets.<br><br>Use of alternative laser sources, e.g., high-performance frequency-tripled or quadrupled solid-state lasers (Nd: YAG).[7]<br><br>Improvements in hardware design and automation of processes
Material Utilization	PLD targets are reusable multiple times.[5]<br>(e.g., more than 20 depositions in the case of $MA_{1-x}FA_xPbI_3$ in [2].	Homogeneous composition through the target thickness after multiple ablations.	In-situ target or film composition analysis to determine when the composition is no longer uniform compared to a fresh target.[5]
Deposition rates	Fast deposition rates can be achieved using high-frequency lasers (> 50 Hz).	Stability and homogenous ablation of high-frequency lasers.<br><br>Current deposition rates of PLD MHPs are at 5 – 10 nm/min.[9] This is the same challenge as co-evaporation.[10]	Developments on new PLD designs (in terms of target and substrate scanning, as well as laser spot sizes) from hardware to software.

References

(1) Smirnov, Y.; Repecaud, P. A.; Tutsch, L.; Florea, I.; Zanoni, K. P. S.; Paliwal, A.; Bolink, H. J.; Cabarrocas, P. R.; Bivour, M.; Morales-Masis, M. Wafer-Scale Pulsed Laser Deposition of ITO for Solar Cells: Reduced Damage vs. Interfacial Resistance. *Mater. Adv.* **2022**, *3*, 3469–3478. https://doi.org/10.1039/d1ma01225h.

(2) Soto-Montero, T.; Kralj, S.; Soltanpoor, W.; Solomon, J. S.; Gómez, J. S.; Zanoni, K. P. S.; Paliwal, A.; Bolink, H. J.; Baeumer, C.; Kentgens, A. P. M.; Morales-Masis, M. Single-Source Vapor-Deposition of $MA_{1-x}FA_xPbI_3$ Perovskite Absorbers for Solar Cells. *Adv. Funct. Mater.* **2023**, 2300588. https://doi.org/10.1002/adfm.202300588.

(3) Ojeda-G-P, A.; Döbeli, M.; Lippert, T. Influence of Plume Properties on Thin Film Composition in Pulsed Laser Deposition. *Adv. Mater. Interfaces* **2018**, *5* (18), 1–16. https://doi.org/10.1002/admi.201701062.

(4) Soto-Montero, T.; Kralj, S.; Rodkey, N.; Gómez, J. S.; Wolffs, J. W.; Kentgens, A. P. M.;

Morales-Masis, M. Quantifying Organic Cation Ratios in Metal Halide Perovskites: Insights from X-ray Photoelectron Spectroscopy and Nuclear Magnetic Resonance Spectroscopy. *Chem. Mater.* **2024**. https://doi.org/10.1021/acs.chemmater.4c00935.

(5) Shepelin, N. A.; Tehrani, Z. P.; Ohannessian, N.; Schneider, C. W.; Pergolesi, D.; Lippert, T. A Practical Guide to Pulsed Laser Deposition. *Chem. Soc. Rev.* **2023**, *52* (7), 2294–2321. https://doi.org/10.1039/d2cs00938b.

(6) Lam Research Introduces Breakthrough Deposition Technique to Enable Next-Generation MEMS for 5G and Beyond https://newsroom.lamresearch.com/2024-03-26-Lam-Research-Introduces-Breakthrough-Deposition-Technique-to-Enable-Next-Generation-MEMS-for-5G-and-Beyond.

(7) Chaluvadi, S. K.; Punathum Chalil, S.; Mazzola, F.; Dolabella, S.; Rajak, P.; Ferrara, M.; Ciancio, R.; Fujii, J.; Panaccione, G.; Rossi, G.; Orgiani, P. Nd:YAG Infrared Laser as a Viable Alternative to Excimer Laser: YBCO Case Study. *Sci. Rep.* **2023**, *13* (1), 1–8. https://doi.org/10.1038/s41598-023-30887-3.

(8) Vakulov, Z.; Khakhulin, D.; Zamburg, E.; Mikhaylichenko, A.; Smirnov, V. A.; Tominov, R.; Klimin, V. S.; Ageev, O. A. Towards Scalable Large-Area Pulsed Laser Deposition. *Materials (Basel).* **2021**, *14* (17). https://doi.org/10.3390/ma14174854.

(9) Soto-Montero, T.; Kralj, S.; Azmi, R.; Reus, M. A.; Solomon, J. S.; Cunha, D. M.; Soltanpoor, W.; Satrio Utomo, D.; Ugur, E.; Vishal, B.; et al. Single-Source Pulsed Laser Deposited Perovskite Solar Cells with >19% Efficiency. *Research Square*. Submission date: 2023-11-29. https//doi.org/10.21203/rs.3.rs-3671187/v1] (accessed 2024-07-11).

(10) Abzieher, T.; Moore, D. T.; Roß, M.; Albrecht, S.; Silvia, J.; Tan, H.; Jeangros, Q.; Ballif, C.; Hoerantner, M. T.; Kim, B.-S.; Bolink, H. J.; Pistor, P.; Goldschmidt, J. C.; Chiang, Y.-H.; Stranks, S. D.; Borchert, J.; McGehee, M. D.; Morales-Masis, M.; Patel, J. B.; Bruno, A.; Paetzold, U. W. Vapor Phase Deposition of Perovskite Photovoltaics: Short Track to Commercialization? *Energy Environ. Sci.* **2024**. https://doi.org/10.1039/D3EE03273F.

(11) Cheng, S.; Chang, Q.; Wang, Z.; Xiao, L.; Chia, E. E. M.; Sun, H. Observation of Net Stimulated Emission in CsPbBr3 Thin Films Prepared by Pulsed Laser Deposition. *Adv. Opt. Mater.* **2021**, *9* (17), 1–9. https://doi.org/10.1002/adom.202100564.

(12) Huang, Y.; Zhou, X.; Zhang, L.; Lin, G.; Xu, M.; Zhao, Y.; Jiao, M.; Zhang, D.; Pan, B.; Zhu, L.; Zhao, F. Tunable Electroluminescence from an N-ZnO/p-GaN Heterojunction with a CsPbBr3interlayer Grown by Pulsed Laser Deposition. *J. Mater. Chem. C* **2020**, *8* (35), 12240–12246. https://doi.org/10.1039/d0tc02807j.

(13) Wang, H.; Wu, Y.; Ma, M.; Dong, S.; Li, Q.; Du, J.; Zhang, H.; Xu, Q. Pulsed Laser Deposition of CsPbBr3 Films for Application in Perovskite Solar Cells. *ACS Appl. Energy Mater.* **2019**, *2* (3), 2305–2312. https://doi.org/10.1021/acsaem.9b00130.

(14) Song, Q.; Zhang, H.; Jin, X.; Wang, H.; Wang, P.; Ijaz, M.; Xu, Q. Highly Stable All-Inorganic CsPbBr3 Perovskite Solar Cells Based on Pulsed Laser Deposition. *Appl. Phys. Lett.* **2023**, *123* (9). https://doi.org/10.1063/5.0156462.

(15) Cesaria, M.; Mazzeo, M.; Quarta, G.; Aziz, M. R.; Nobile, C.; Carallo, S.; Martino, M.; Calcagnile, L.; Caricato, A. P. Pulsed Laser Deposition of Cspbbr3 Films: Impact of the Composition of the Target and Mass Distribution in the Plasma Plume. *Nanomaterials* **2021**, *11* (12). https://doi.org/10.3390/nano11123210.

(16) Hu, J.; Wang, X.; Lin, L.; Xu, J.; Liu, M.; Wang, R.; Li, X.; Tao, L.; Sui, Y.; Song, B. High-Performance Self-Powered Photodetector Based on the Lateral Photovoltaic Effect of All-Inorganic Perovskite CsPbBr3 Heterojunctions. *ACS Appl. Mater. Interfaces* **2023**, *15* (1), 1505–1512. https://doi.org/10.1021/ACSAMI.2C16347.

(17) Huang, Y.; Zhang, L.; Wang, J.; Zhang, B.; Xin, L.; Niu, S.; Zhao, Y.; Xu, M.; Chu, X.; Zhang, D.; Qu, C.; Zhao, F. Growth and Optoelectronic Application of CsPbBr 3 Thin Films Deposited by Pulsed-Laser Deposition . *Opt. Lett.* **2019**, *44* (8), 1908. https://doi.org/10.1364/ol.44.001908.

(18) Marra, M.; Provenzano, C.; Cesaria, M.; Cataldo, R.; Monteduro, A. G.; Caricato, A. P. CsPbBr3 Films Grown by Pulsed Laser Deposition: Impact of Oxygen on Morphological Evolution and Properties. *Processes* **2023**, *11* (9), 1–17. https://doi.org/10.3390/pr11092514.

(19) Zhou, Y.; Yuan, B.; Wei, H.; Xu, F.; Li, Y.; Chen, X.; Cao, B. Stable CsPbX3mixed Halide Alloyed Epitaxial Films Prepared by Pulsed Laser Deposition. *Appl. Phys. Lett.* **2022**, *120* (11). https://doi.org/10.1063/5.0081955.

(20) Kiyek, V. M.; Birkhölzer, Y. A.; Smirnov, Y.; Ledinsky, M.; Remes, Z.; Momand, J.; Kooi, B. J.; Koster, G.; Rijnders, G.; Morales-Masis, M. Single-Source, Solvent-Free, Room Temperature Deposition of Black γ-CsSnI3 Films. *Adv. Mater. Interfaces* **2020**. https://doi.org/10.1002/admi.202000162.

(21) Sebastiá-Luna, P.; Rodkey, N.; Mirza, A. S.; Mertens, S.; Lal, S.; Carranza, G. A. M.; Calbo, J.; Righetto, M.; Sessolo, M.; Herz, L. M.; Vandewal, K.; Ortí, E.; Morales-Masis, M.; Bolink*, H. J.; Francisco*, P. Chalcohalide Antiperovskite Thin Films with Visible Light Absorption and High Charge-Carrier Mobility Processed by Solvent-Free and Low-Temperature Methods. *Chem. Mater.* **2023**, *35* (16), 6482–6490.

(22) Rodkey, N.; Kaal, S.; Sebastia-Luna, P.; Birkhölzer, Y. A.; Ledinsky, M.; Palazon, F.; Bolink, H. J.; Morales-Masis, M. Pulsed Laser Deposition of Cs2AgBiBr6: From Mechanochemically Synthesized Powders to Dry, Single-Step Deposition. *Chem. Mater.* **2021**, *33* (18), 7417–7422. https://doi.org/10.1021/acs.chemmater.1c02054.

(23) Bansode, U.; Naphade, R.; Game, O.; Agarkar, S.; Ogale, S. Hybrid Perovskite Films by a New Variant of Pulsed Excimer Laser Deposition: A Roomerature Dry Process. *J. Phys. Chem. C* **2015**, *119* (17), 9177–9185. https://doi.org/10.1021/acs.jpcc.5b02561.

(24) Bansode, U.; Ogale, S. On-Axis Pulsed Laser Deposition of Hybrid Perovskite Films for Solar Cell and Broadband Photo-Sensor Applications. *J. Appl. Phys.* **2017**, *121* (13). https://doi.org/10.1063/1.4979865.

(25) Soto-Montero, T.; Soltanpoor, W.; Kralj, S.; Birkhölzer, Y. A.; Remes, Z.; Rijnders, G.; Morales-masis, M. Single-Source Pulsed Laser Deposition of MAPbI3. *2021 IEEE 48th Photovolt. Spec. Conf.* **2021**, 1318–1323. https://doi.org/10.1109/PVSC43889.2021.9518799.

(26) Dunlap-Shohl, W. A.; Barraza, E. T.; Barrette, A.; Gundogdu, K.; Stiff-Roberts, A. D.; Mitzi, D. B. MAPbI3 Solar Cells with Absorber Deposited by Resonant Infrared Matrix-Assisted Pulsed Laser Evaporation. *ACS Energy Lett.* **2018**, *3* (2), 270–275. https://doi.org/10.1021/acsenergylett.7b01144.

(27) Solomon Solomon Sathiaraj, J. S.; Al., E. Room Temperature Epitaxy of CH3NH3PbI3 Halide Perovskite by Pulsed Laser Deposition. *Research Square.* Submission date*:* 2023-12-18. https://doi.org/10.21203/rs.3.rs-3730125/v1 (accessed 2024-07-11).

(28) Ismail, R. A.; Abdulnabi, R. K.; Abdulrazzaq, O. A.; Jawad, M. F. Preparation of MAPbI3 Perovskite Film by Pulsed Laser Deposition for High-Performance Silicon-Based Heterojunction Photodetector. *Opt. Mater. (Amst).* **2022**, *126* (March), 112147. https://doi.org/10.1016/j.optmat.2022.112147.

(29) Barraza, E. T.; Stiff-Roberts, A. D. Phenomenological Mechanisms of Hybrid Organic–Inorganic Perovskite Thin Film Deposition by RIR-MAPLE. *J. Appl. Phys.* **2020**, *128* (10), 105303. https://doi.org/10.1063/5.0015962.

(30) Liu, D.; Li, X.; Shi, C.; Liang, Q. CH3NH3PbI3 Thin Films Prepared by Pulsed Laser Deposition for Optoelectronic Applications. *Mater. Lett.* **2017**, *188* (October 2016), 271–274.

https://doi.org/10.1016/j.matlet.2016.10.113.

(31) Miyadera, T.; Sugita, T.; Tampo, H.; Matsubara, K.; Chikamatsu, M. Highly Controlled Codeposition Rate of Organolead Halide Perovskite by Laser Evaporation Method. *ACS Appl. Mater. Interfaces* **2016**, *8* (39), 26013–26018. https://doi.org/10.1021/acsami.6b07837.

(32) Jasti, N. P.; Tirosh, S.; Halder, A.; Teblum, E.; Cahen, D. Continuous Wave Laser-Assisted Evaporation of Halide Perovskite Thin Films from a Single Stoichiometric Source. *J. Vac. Sci. Technol. A* **2024**, *42*, 043406. https://doi.org/10.1116/6.0003607.

(33) Hoffmann-Urlaub, S.; Zhang, Y.; Wang, Z.; Kressdorf, B.; Meyer, T. Fabrication of Tin-Based Halide Perovskites by Pulsed Laser Deposition. *Appl. Phys. A Mater. Sci. Process.* **2020**, *126* (7), 1–11. https://doi.org/10.1007/s00339-020-03699-9.
